# Developing a novel claim‐based algorithm to identify incident and prevalent dementia using Medicare claims of the Atherosclerosis Risk in Communities (ARIC) cohort

**DOI:** 10.1002/alz.089988

**Published:** 2025-01-09

**Authors:** Tiansheng Wang, Virginia Pate, Dae Kim, Melinda C Power, Gwenn A Garden, Priya Palta, David S. Knopman, Michelle Jonson‐Funk, Til Stürmer, Anna M. Kucharska‐Newton

**Affiliations:** ^1^ Universtiy of North Carolina at Chapel Hill, Chapel Hill, NC USA; ^2^ University of North Carolina, Chapel Hill, NC USA; ^3^ Hebrew SeniorLife Marcus Institute for Aging Research, Boston, MA USA; ^4^ The George Washington University, Washington, DC USA; ^5^ University of North Carolina Chapel Hill, Chapel Hill, NC USA; ^6^ Mayo Clinic, Rochester, MN USA; ^7^ University of North Carolina at Chapel Hill, Chapel Hill, NC USA; ^8^ University of North Carolina Gillings School of Global Public Health, Chapel Hill, NC USA

## Abstract

**Background:**

Pharmacoepidemiologic studies assessing drug effectiveness for Alzheimer’s disease and related dementias (ADRD) are increasingly popular given the critical need for effective therapies for ADRD. To meet the urgent need for robust dementia ascertainment from real‐world data, we aimed to develop a novel algorithm for identifying incident and prevalent dementia in claims.

**Method:**

We developed algorithm candidates by different timing/frequency of dementia diagnosis/treatment to identify dementia from inpatient/outpatient/prescription claims for 6,515 and 3,997 participants from Visits 5 (2011‐2013; mean age 75.8 (SD 5.3) years) and 6 (2016‐2017; mean age 79.8 (SD 4.8) years) of the ARIC cohort with ≥ 1‐year continuous Medicare Part A & B enrollment prior to each visit. We evaluated algorithm performance (sensitivity/specificity/positive predictive value (PPV)/negative predictive value (NPV)) against the gold standard of the ARIC dementia classification based on an extensive battery of neurocognitive tests administered at each visit. We compared performance of the new algorithm with that of three existing algorithms (Jain, Bynum, Lee) and their relaxed forms (4‐year window, dementia diagnosis plus a depression diagnosis or dementia evaluation procedure, ignoring code position) to identify incident (n = 90 at V6) and prevalent dementia (n = 190 at V5; n = 160 at V6).

**Result:**

The newly developed primary algorithm requires presence of a dementia diagnosis code followed by another diagnosis code within 1 year (under condition that the following non‐specific ICD‐9 diagnostic codes, 290.40, 290.41, 290.11, 290.3, should appear no more than once), or a subsequent dementia treatment prescription within 90 days (requiring Medicare Part D enrollment ≥ 6 months). The PPV of the new algorithm in the identification of incident dementia was PPV of 72.7%, with a specificity of 99.1%, outperforming existing algorithms. Its sensitivity of 38.7% and NPV of 96.2% were comparable to those achieved by existing algorithms. Sensitivity analysis restricting to ICD‐10 diagnostic codes increased the PPV and attenuated its sensitivity by 6%. Similar comparative performance was observed in the identification of prevalent dementia.

**Conclusion:**

A novel algorithm for identifying dementia from Medicare claims, developed using linked ARIC‐Medicare data, outperforms previous algorithms and stands as a valuable tool in the assessment of ADRD drugs from real‐world data.

